# A Secure and Scalable Smart Home Gateway to Bridge Technology Fragmentation

**DOI:** 10.3390/s21113587

**Published:** 2021-05-21

**Authors:** Ezequiel Simeoni, Eugenio Gaeta, Rebeca I. García-Betances, Dave Raggett, Alejandro M. Medrano-Gil, Diego F. Carvajal-Flores, Giuseppe Fico, María Fernanda Cabrera-Umpiérrez, María Teresa Arredondo Waldmeyer

**Affiliations:** 1 Life Supporting Technologies (LifeSTech), ETSI Telecomunicaciones Universidad Politécnica de Madrid, Av. Complutense s/n, 28040 Madrid, Spain; eugenio.gaeta@lst.tfo.upm.es (E.G.); rgarcia@lst.tfo.upm.es (R.I.G.-B.); amedrano@lst.tfo.upm.es (A.M.M.-G.); dcarvajal@lst.tfo.upm.es (D.F.C.-F.); gfico@lst.tfo.upm.es (G.F.); chiqui@lst.tfo.upm.es (M.F.C.-U.); mta@lst.tfo.upm.es (M.T.A.W.); 2 W3C/ERCIM, 2004, Route des Lucioles, Sophia Antipolis, 06410 Biot, France; dsr@w3.org

**Keywords:** Internet of Things, smart environment, web of things, semantics, gateway, technology fragmentation

## Abstract

Internet of Things (IoT) technologies are already playing an important role in our daily activities as we use them and rely on them to increase our abilities, connectivity, productivity and quality of life. However, there are still obstacles to achieving a unique interface able to transfer full control to users given the diversity of protocols, properties and specifications in the varied IoT ecosystem. Particularly for the case of home automation systems, there is a high degree of fragmentation that limits interoperability, increasing the complexity and costs of developments and holding back their real potential of positively impacting users. In this article, we propose implementing W3C’s Web of Things Standard supported by home automation ontologies, such as SAREF and UniversAAL, to deploy the Living Lab Gateway that allows users to consume all IoT devices from a smart home, including those physically wired and using KNX^®^ technology. This work, developed under the framework of the EC funded Plan4Act project, includes relevant features such as security, authentication and authorization provision, dynamic configuration and injection of devices, and devices abstraction and mapping into ontologies. Its deployment is explained in two scenarios to show the achieved technology’s degree of integration, the code simplicity for developers and the system’s scalability: one consisted of external hardware interfacing with the smart home, and the other of the injection of a new sensing device. A test was executed providing metrics that indicate that the Living Lab Gateway is competitive in terms of response performance.

## 1. Introduction

The Internet of Things (IoT) refers to the grouping and interconnection of devices and objects through a network, where they can all be visible, interact and exchange data. The objects or devices can be of a wide range of types, from sensors and mechanical devices to everyday objects such as a refrigerator or a light bulb [1]. IoT technologies allow the development of a wide diversity of products, useful in contexts such as cities, home or health services [2]. One of the most common uses of IoT technology is home automation, however, both consumers and developers suffer from issues related to the high degree of technology fragmentation, given that it limits interoperability, increases development costs holding back IoT’s true potential. A home automation system (HAS) is a set of networked smart devices used to automate tasks in a home environment. In the last ten years, the HAS market has grown significantly, therefore, to support smart home applications, a multitude of technologies have been developed [3,4]. In essence, better user-friendly interfaces have been adopted, such as voice command interaction using smart speakers (e.g., Amazon Echo) [5]. The advent of these devices has offered improvements in usability along with applications for entertainment, news, and home shopping, although a unique tool that performs as the “master control” and allows users to easily interact with all these devices does not exist yet. To build products that address multiple proprietary ecosystems, as to reach the critical market mass for return of investment, developers need to master the many different technologies and approaches adopted by different companies [4]. This fragmentation needs to be addressed as much of the potential will be in realising high value services that combine heterogeneous sensors, actuators and multiple sources of information. Moreover, fragmentation is underlined by a lack of interoperability across proprietary technologies, and a profusion of incompatible specifications from different standards development organisations [6]. The mitigation of the problems associated with fragmentation require the use of standards to define how smart devices communicate and interact with each other. In turn, these standards must be supported by a large community of developers, to ensure that constant efforts are focused at increasing interoperability. Thus, the World Wide Web Consortium (W3C) seeks to reduce the lack of interoperability through an abstraction layer that reduces the effort needed by developers to cover multiple systems and IoT standards. ‘Web of Things’ (WoT) [6] is W3C’s novel standard that focuses on digital twins for physical and abstract things. According to the concept of digital twins, each “thing” has a uniform resource identifier (URI) that is used to access “Thing Descriptions”, expressed in terms of the Resource Description Framework (RDF) [7] and serialised as JavaScript Object Notation for Linked Data (JSON-LD) [8]. Since the appearance of the semantic web in recent years, the ontology concept has received great attention. Basically, it conceptualizes and organizes semantic information of application domains. In addition, its use extends to Service Oriented Computing (SOC) to facilitate discovery and composition of smarter services [9]. Through semantic technologies it is possible to have a higher level of interoperability and, by describing resources (i.e., data, actuators, sensors, etc.) and devices with their conceptual meaning, also it is possible to create abstract services which transcends their technical implementation.

The WoT greatly facilitates service composition by decoupling applications from the details needed to access servers that expose things. For instance, one server might expose things using the oneM2M standards [10], whilst another might instead use the standards from the Open Connectivity Foundation (OCF) [11]. Both servers may use the same underlying protocol, e.g., HTTP (Hypertext Transfer Protocol), but in incompatible ways. The WoT further allows applications to access and reason with the metadata for services using RDF as a common framework, independently of the underlying IoT systems, allowing to expose the things included in the Thing Descriptor (TD), which is essentially a list of all devices included. The TD is based on an interaction model that can support diverse messaging paradigms and implements its own Interaction Patterns as the following:

- Properties are readable/writable data points.
- Actions are callable processes.
- Events are asynchronous interactions that enable to push data. This is how the WoT copes with the network-facing APIs from most IoT Platforms.

In this way, the abstract layer is then capable of applying reasoning allowing for smarter management, retrieval and combination of resources and services utilizing the different implementations even if these are incompatible solutions. The system is embedded with sufficient intelligence to find the most appropriate abstract service to invoke, sometimes resulting in service composition to achieve the requested goal. These are only the basics of Semantic Interoperability. However, the generic algorithms need to rely on formal definitions of the different knowledge domains, these definitions are ontologies. Ontologies, such as Smart Appliances Reference (SAREF), provide the specific knowledge required for smart environments, and thus can improve interoperability and integration [9,12] addressing also the high degree of fragmentation [13] that we observe nowadays in home automation.

Additionally, when developing and deploying a HAS, a complex aspect to solve is security, as it is critical for any interconnected digital asset manager. Private companies [14], public bodies [15,16] and standardization and harmonization institutes (e.g., RFC 2196 Site Security Handbook), have published recommendations aimed at improving the quality and consistency of the cybersecurity across interconnected systems. Such recommendations are addressed towards system managers, organization officers, service providers, infrastructure owners, product manufacturers, developers, and end users. Generally, these sets of recommendations detail diverse aspects, but generally converge on similar rules and guidelines. They state that cybersecurity should be a continuous process, a self-improvement procedure that excecises cybersecurity threats and measures evaluation.

### 1.1. Related Work

Similar solutions aimed at dealing with IoT devices and ecosystems’s interoperability have already been deployed, but most of them lack the integration of coexisting systems, such as home automation systems controlling physical installations.

A Raspberry Pi controller for wired devices presented in [17] implemented a gateway allowing access to devices connected to a KNX^®^ network in a Web-of-Things manner. The complexity of the architecture and the cost of processing XSLT files makes this approach not applicable with such low computational resource. One clear limitation of this proposition is that it depends on the a DNS server access, in most cases restricted by security policies. Also, the security in this deployment does not contemplate user authentication.

The eWot [18] centralises the storage of all things descriptions (IoT Profiles). The IoT devices managed by eWot comprise different APIs, formats, and models and interoperability is addressed by a SPARQL-query-based mechanism to manage devices.

Another approach, the IoT-based Semantic Interoperability Model (IoT-SIM) [19], tackles interoperability among healthcare IoT devices, through which physicians can communicate with their patients and monitor their current health status. IoT devices and their data are managed by Sensor Web Enablement (SWE) to manage and consume IoT devices. Through this external service, heterogeneity between devices is addressed.

An interesting solution also implemented in Smart Homes [20] is a semantic model for smart objects that implements ontologies and description logics, enabling intelligent functions, reasoning over service data and interoperability. Also others have shown how to provide a gateway that translates HTTP instructions from clients into KNX^®^ instructions to home devices [17].

Additionally, the Semantic Gateway as Service (SGS) [21] addresses interoperability through gateway and Semantic Web enabled IoT architecture, allowing to exchange messaging protocols (e.g., XMPP, CoAP, and MQTT). SGS architecture establishes external connections with the gateway through the mentioned protocols and then it connects via REST or pubsub to, for instance, a cloud service or other SGSs. Sensor data is annotated using domain specific ontologies and converted to JSON format to comply with RESTful protocols.

To the best of our knowledge, topics such as Security, Scalability and trust are not addressed as part of the challenge of overcoming technology fragmentation. Such features are key elements to include when developing a home devices management system, especially if multiple users are going to make use of it in diverse scenarios.

The motivation to develop the solution presented in this work raises from Horizon 2020 project Plan4Act (N° 732,266). In this project, the wireless transmission and real-time decoding of rhesus macaques’ brain signals to convert them into smart home devices’ commands is pursued. In this context, it was requested a secure and trusted gateway that listens to a monkey’s brain signals neural decoder and executes commands at the Smart House. For time and effort saving, little to no effort of interfacing the experimental setting with a domotic environment control system was required. Thus, in this work we propose the Living Lab Gateway (from now on, LLG) that deals with the lack of interoperability and the technologies fragmentation at web level. A scalable WoT interface for home automation services was built, where devices are semantically described through standard vocabularies and ontologies, while providing security and trust features.

### 1.2. Basic Idea

The main purpose of our development is to provide a solution for interfacing with an ecosystem of devices (in this case a Smart Home) that contains several different technologies. In a nutshell, the LLG provides a secure access layer that offers an abstract description of every device, which is independent from their internal denomination, physical location and communication protocol. Thus, it offers a unified and simplified method to access and consume these, reducing the coding effort for developers and allowing to easily populate the ecosystem with new devices. Moreover, security and scalability design criteria were considered when developing and deploying this solution. A key feature this development includes is its adaptability to a wide range of technology domains, as it only takes to include the ontologies under which these are defined into the device’s description in order to make them available, simplifying the LLG’s re-purposing.

### 1.3. Contribution

In this work we contribute to mitigate technology fragmentation and provide better accessibility to device manipulation for heterogeneous device ecosystems, even in a more human-understandable format for non-expert developers. Moreover, we include novel WoT standard and articulate it with semantic content to merge multiple technology domains technology use through a unique tool. We also provide metrics and the corresponding analysis regarding the LLG benefits and competitiveness.

The next sections are organized as follows: in Section 2 methodology and materials are described. In Section 3, implementation results and tests are presented. In Section 4, discussion, lessons learnt and future steps. Section 5, conclusions.

## 2. Materials and Methods

This section is addressing the materials and methods used in order to deploy and test the LLG, implementing interoperability, seamless connectivity of heterogenous devices, security, scalability, resilience, authentication and authorization.

### 2.1. Materials

#### 2.1.1. Containers and Orchestration Technologies

Containers and orchestration technologies are used for the deployment of a scalable and resilient infrastructure for the LLG. A container (e.g., Docker) is an isolated, closed, and lightweight environment where a piece of software is running. It runs on the host machine’s operating system providing a higher degree of flexibility compared to hypervision technologies (e.g., Virtual Machines). Due to this high degree of flexibility, containers are the best option for microservices implementation. Moreover, when the objective is to implement microservices-based architectures, we need complementary technologies that provide more reliability, security, scalability, resource optimization and automation. These are called orchestration technologies and Kubernetes (commonly referred to as “K8s”) [22] was our choice in this work. K8s is an open-source platform for automating the deployment, scaling, and containerized applications management, which distributes containers into pods across multiple nodes that, ultimately, form clusters. Key elements of Kubernetes are Kubernetes Pods, Kubernetes Nodes, Master node of kubernetes, Kubernetes cluster and Load Balancers. In this paper we will describe how to provide an implementation that maps these devices within a secure, adaptable, scalable and resilient infrastructure based on semantics and WoT.

#### 2.1.2. Smart House Living Lab

The Smart House Living lab (SHLL) is an automated environment where technological solutions can be tested and validated with real users. It contains devices and appliances both IoT and wired, and it is a fully adaptable domotic empowered open space that can be configured at runtime on the users‘ needs: one day it can represent a house, the day after a ward of a hospital or an office. The Information Technology (IT) infrastructure available at the Smart House Living Lab includes a KNX^®^ twisted pair deployment which integrates almost all the devices available at Living Lab such as doors, windows, blinds, sensors, air conditioner and media devices. It is worth mentioning one device in particular: the Smart Cabinet, which is an IoT condiment dispenser fully integrated to the SHLL developed at UPM which is the Plan4Act’s neural decoder target.

#### 2.1.3. MongoDB Database

MongoDB [23] is a general purpose, distributed, document-based database, that supports different JSON documents: JSON, BSON, XML and BLOBs. Queries are JSON, and thus easily composable, overcoming SQL queries. It uses a powerful query language that enables field filtering and sorting, bringing solutions to more complex use-cases [24]. MongoDB is used to store the Thing Description generated for the devices installed in the SHLL and to track their state changes in a consistent way, allowing scalability and better performance in the management of data in JSON format.

#### 2.1.4. Structured and Linked Data Format

JSON-LD (JSON for Linked Data) [8] is a media type that enables the above-mentioned layer 3 style of the REST architectural style and data self-description. The support of JSON-LD data linking in WoT allows the LLG to address standardization and interoperability over different domains of interest. In our solution we address the Smart Home domains translating the universAAL device ontology [25] with the standard SAREF ontology [26]. Structured and linked data format is used for describing the data and to link their meaning with standard vocabularies and ontologies within the interfaces of the LLG.

#### 2.1.5. Web of Things

The WoT [6] is the standard that has been adopted in order to describe the interfaces that allow client to interact with the devices of the Living Lab. The Thing Description (TD) is a central WoT element and consists of 5 important elements: semantic metadata, an interaction model (properties, actions and events which were already described), a semantic schema, a security schema and web linking features to establish relations between Things. They are also serialized to JSON-LD by default and allows semantics to be machine-understandable and confers better usability for developers [27].

In this work, the WoT is used to add semantic meaning to the KNX^®^ devices that are installed in the lab. Each service and device within the SHLL is defined as “Things” and are mapped on a Thing Description, compliant with WoT. Each Thing has a URI that is used to access descriptions of things expressed in terms of RDF (Resource Description Framework) and serialised as JSON-LD. Thing descriptions include:

- The interaction model exposed to client applications in terms of properties, actions and events, along with the associated data models and metadata, such as units of measure.
- Semantic descriptions of the types of things and the context in which they are situated, e.g., a temperature sensor for a given room in a house, expressed using the ontologies agreed between the suppliers and consumers of services.
- Communications metadata that describes how the client platform can access things exposed by a server platform.
- Security metadata describing the requirements for a secure access to the thing.

#### 2.1.6. Microservices

A microservice [28] supports interoperability through message-based communication and a microservice architecture is based on scalable and evolvable software systems. Most valuable assets of microservices are their speed, safety and scalability. The aim of the microservice architecture was to replace monolithic applications with systems composed of multiple exchangeable components [29]. In order to implement a reliable infrastructure for the LLG, on the top of containerization and orchestration technology, an architectural pattern was designed for the infrastructure to be fault-tolerant and to allow scalability. Such pattern is the microservice based infrastructure.

#### 2.1.7. Three-Tier Architecture

As well as for the infrastructure, the LLG solution needs certain degrees of flexibility and separability, achieved by grouping components in layers. The LLG architecture follows a three-tier pattern. The topological distribution of an application modules can change depending on the system requirements. The above-mentioned tiers are logical layers, and can be described as follows: a Presentation Tier, a Middle Tier and a Data Tier. The presentation tier (i.e., user services layer) provides access to traditional application and Web-based applications and presents data, allowing its manipulation and new data entry. The middle tier is the business services or logic layer. Its components can be allocated on a server machine and are designed to keep data structure consistency across diverse databases. Not being of exclusive use for a specific client, middle-tier components can be used by all applications and move around different locations depending on each instance. The data tier, or data services layer, interacts specifically with data, acting as database access manager and it consists of data access components to enable resource sharing [30].

#### 2.1.8. REST Interfaces

The interaction between the LLG service layer and third parties is provided through REpresentational State Transfer (REST), which is a web-based architecture. A key component to be defined when talking about REST is the concept of resources (e.g., a device). Different representations of this device are created (by the server) on demand, allowing clients to ask for a representation of a Device. This representation can be served to clients in the format they requested (JSON, for instance) even though internally it may be stored in a different format. On the other hand, methods in REST are invariant (in terms of behaviour across devices), and the HTTP specification defines which methods that can be used (e.g., GET to retrieve a resource and POST to create one). The use of standard textual formats brings flexibility related to the use of resources, being XML and JSON formats the most popular of those running on HTTP [31].

In a hypermedia format, hypermedia controls represent protocol information. A hypermedia control includes the address of a linked resource, together with some semantic markup. In the context of the current resource representation, the semantic markup indicates the meaning of the linked resource. The acronym “hypermedia as the engine of application state” (HATEOAS) [31] describes the main principles of the REST architectural style.

#### 2.1.9. Stateless Oriented Services

LLG services can be understood in isolation. There is no stored knowledge of or reference to past transactions. Each transaction is made as if from scratch for the first time in a stateless manner. Both RESTful and Hybrid RESTful service rely on HTTP. This means that they are missing a security. In order to maintain the confidentiality and integrity of resource representations is quite recommended to use TLS [32] and make resources accessible over a server configured to serve requests only using HTTPS.

With HTTPS (HTTP over TLS) we are solving confidentiality and integrity but we are still missing another fundamental aspect of security that is authentication. Users or services interact with the application in what is called a session, defined as the series of interactions they perform during the interaction period. A stateless application [33] is defined as an application that is agnostic or has no record of previous interactions with clients or their outcomes. They can scale horizontally since any request can be serviced by any resource (e.g., EC2 instances, Google cloud functions, AWS Lambda functions). A stateless service implies a stateless authentication, thus, at server side, the state of a user is not maintained, and the server is completely unaware of who sends the request. We can achieve the stateless authentication by using JWT (JSON Web Token), which is an example of a token-based approach [34]. An advantage of token based approach is that not even ID data from previous sessions are needed, so they are not stored.

### 2.2. Methods

The LLG was implemented and tested in different modalities aiming to cover all features and functions of our solution. Its main functionalities and features were tested in two different scenarios.

#### 2.2.1. Implementation Scenarios

**Figure 1:**
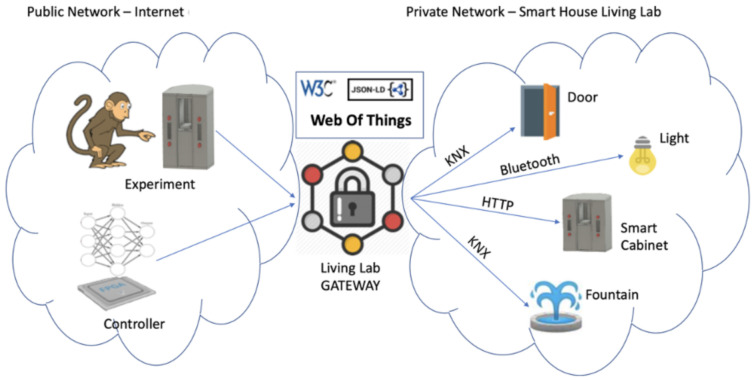
Living Lab Gateway solution for Plan4Act’s demo 1.

Scenario 1 (addressing Plan4Act project’s needs) is the remote control of living lab devices by a hardware embedded classifier of neural data extracted from monkeys in real time. This is Plan4Act project’s Demo 1 and comprises a variant in which the house control can be executed remotely from an interface located in an experimental protocol employing Rhesus macaques. This scenario is illustrated in Figure 1 below:

In this scenario, a Field Programmable Gate Array controller (where the mentioned neural controller is embedded) commandedthe SHLL devices (both IoT and KNX^®^ devices) through the LLG. Sequences of commands are sent by the controller in two modalities: sequential mode and proactive mode. The first, consists in emitting commands one by one (e.g., open the main door and, once this action is done, turn the lights on). On the other hand, the latter comprises sets of commands into a unique one, executing all actions in one step to produce a time gain. Time gain associated to proactive control is the main Plan4Act project’s goal (e.g., open the main door, turn the lights on and open the bathroom door at the same time).

On the other hand, in scenario 2, an external air quality management system is injected into the SHLL ecosystem (i.e., added to the Thing Descriptor) and operates the Smart House devices (e.g., doors, windows and smoke extractor) whenever a high concentration of air pollutants is detected [35]. In this case, our solution was tested against technology fragmentation by introducing a novel device (the air quality sensor) based on a proprietary interface is exposing a preset API.

The LLG’s feature demonstration in each scenario described above can be summarized as follows:

- The infrastructure deployment is explained and tested under Scenario 1 conditions.
- Scenario 1 allowed to test the interaction protocol for users‘authentication and authorization to access devices (security, authentication and authorization features).
- In Scenario 1, devices abstraction and mapping into ontologies were demonstrated, going from datapoints to Things and, finally, to ontologies.
- In Scenario 2, dynamic configuration and injection of devices is demonstrated. The procedure to add novel devices and include them into the environment of home devices is shown.

#### 2.2.2. Response Time Comparison Test

In order to evaluate the LLG’s performance, we tested the round-trip time that elapsed to change the Smart Cabinet’s status (one of the SHLL’s devices) by executing a direct HTTP request and also through the LLG. The Smart Cabinet is one of the elements developed during Plan4Act project and the authors have access to the firmware code, which simplifies its use for testing.

The objective is to perform a paired test comparison to find out whether the mean response time elapsed by the LLG is not significantly different of the elapsed by a direct request. For this test, the following parameters will be considered:

1. Local execution time, being this the time the Smart Cabinet needs since it receives a request until the command is executed (measurement taken via firmware).
2. Local network execution, a direct HTTP interface with the device that is accessible on the local network;
3. Secure remote execution, using the LLG to send command to the devices.

#### 2.2.3. Cybersecurity Model

**Figure 2:**
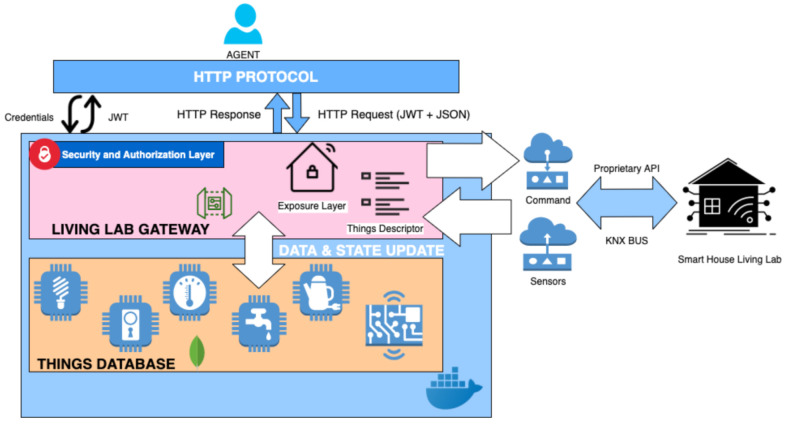
Living Lab Gateway Architecture.

For our cybersecurity model and self auditing method we have selected the STRIDE and DREAD models as basis [36]. We will first Identify the assets that need protection from cyber-attacks, being this list of assets extracted directly from the architecture elements (see Figure 2). Once the List of assets are identified, the STRIDE model will be applied to identify the threats. The STRIDE model identifies 6 classes of threats: Spoofing, Tampering, Repudiation, Information disclosure, Denial of service, and Elevation of Privilege. Once Threats have been identified, the next step is to analyse the Risk they represent, and for this we use the DREAD model. DREAD model identifies indicators to assess the threat impact: Damage Potential, Reproducibility, Exploitability, Affected users, Discoverability. We classify each Thread identified with a score on each indicator as Low (L), Medium (M), or High (H) in terms of its impact on our system and/or each threat’s probability of occurrence. Finally, based on the identified threats and their risk analysis, we propose and mitigation actions. These actions can be organizational, physical, hardware, software or networking measures. For threats with lower risks we also note that in case the counter-measures fail the risks can be accepted.

## 3. Results

In this section, the implementation of the LLG’s service infrastructure is addressed, this is, the microservices architecture based on a RESTful approach, security and authentication for stateless service.

The LLG has a three-layer infrastructure. The first layer represents the physical KNX^®^ infrastructure that connects all the devices. The second layer is a critical point of access because it maps the KNX^®^ datapoints into IP datagrams and it does not provide security, yet its access is blocked by a firewall when the connection is made from inside the Lab. In order to provide access from outside the lab a whitelist is set up allowing communication only from the LLG at the third layer. Layers 1 and 2 are in the same living lab installation while layer 3 is in the cloud. The Calimero Library [37] provides a discovery feature that is used for scanning KNX^®^ servers and datapoints, a feature that is used by the LLG to populate a MongoDB database.

**Figure 3:**
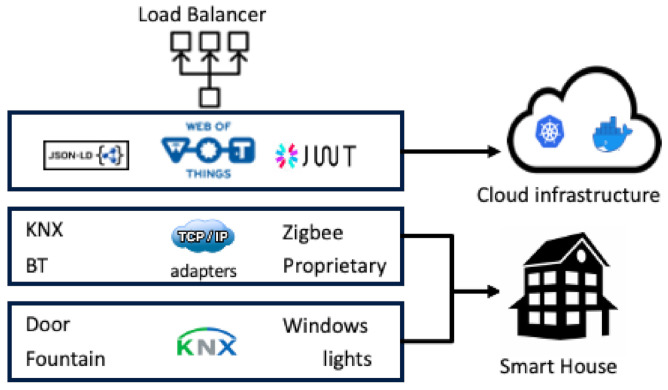
Three layer Architecture description.

As shown in Figure 3 below, the physical KNX^®^ infrastructure and the KNX^®^-IP bridge are installed on the SHLL, while the LLG relies on a cloud infrastructure. This layer is deployed using container and orchestrator technologies. Two Docker containers and a cloud file system are orchestrated within a Kubernetes cluster. The first Docker container is mapped on a cloud load balancer Kubernetes service that manages a Java backend application, while a MongoDB Docker container, connected with a network file system on the cloud provider, manages the data representing the devices’ WoT interfaces. This configuration of the Kubernetes cluster allows a high availability and reliability for (a) the entry point service (the Java backend application); (b) the WoT interfaces of the devices (the MongoDB container); and (c) decoupling between hardware and software components of the SHLL.

A more detailed description of the implementation is shown in Figure 2, where the complete information flow, actors and technologies are depicted.

### 3.1. Dynamic ConfiGuration and Injection of Devices

**Figure 4:**
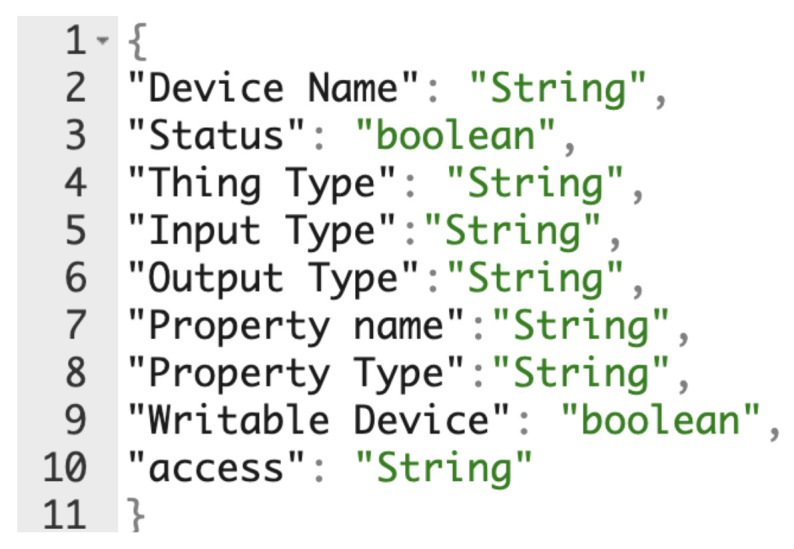
Required JSON for Device addition to TD.

The LLG provides a functionality that builds automatically a Thing Description (TD) either through an API (collecting a list of devices from a database or allowing the injection of new devices) or by listening to the KNX^®^’s bus, thus creating the internal database of the available devices of the LLG. The database can be privately edited in order to add also additional information to the devices such as description, name, properties, data types and so on. However, this functionality is not publicly available in the main TD of the living lab. This feature allows to modify a device on demand, enabling the user to change the current configuration of the lab. For instance, if it is needed to arrange the SHLL configuration to represent an office, it would be possible to redefine the door as office door, the living room as meeting room and so on. Through Calimero library, it is also possible to “listen” when a new datapoint is added to KNX^®^ BUS or when a value of a datapoint is written on the BUS. The LLG is constantly checking on these events and maintains consistency between the MongoDB database and the current state of KNX^®^ devices. Moreover, it supports some functionalities similar to Amazon Alexa or Google Home, as new devices can be connected through third party APIs. Thus, it is possible to inject other non-KNX^®^ devices in a common infrastructure. A key endpoint that is used to populate the list of devices with external IoT devices is /add endpoint. This feature requires the user to perform an HTTP PUT request including the JSON Schema in the code snippet shown in Figure 4 below.

This request will add a TD of the device into the local database, including its properties and interactions, as defined by the information provided and making it accessible from the devices list. An example of the application of this feature is found in [35], which also provides more details regarding interfacing simplicity with the SHLL and LLG tests event log.

### 3.2. Providing Security, Authentication and Authorization

Following the STRIDE method, we identified the assets that need to be protected. In almost all instances, these are derived from he architecture, e.g., the Thing Description, the LLG itself, the physical devices and, even for completeness, we included important infrastructure assets such as the SHLL’s infrastructure. After all, if attackers is able to open the laboratory door, then they would have breached the SHLL and everything in it.

Table 1 summarizes the threats identified in each of the STRIDE model’s classes. For some assets, their associated cybersecurity threats are entirely dependent on other assets, and we declare these threats as implemented “through” the critical asset. This aides in applying the STRIDE method, as these possible vulnerabilities are contextualized exclusively to the asset itself, and therefore staying focused on the asset under analysis each time, while also being complete in the analysis.

Once the threats are identified, their DREAD classification is determined, and for each given threat, a mitigation action is assigned. Table 2 shows this analysis. As it can be seen, most actions correspond with a organizational process placed to avoid the threat, for example placing physical barriers, performing reviews, backups and updates.

The mitigation action of Controlled Network management, is defined using the Open Systems Interconnection (OSI) reference model [38].

At Data link layer (OSI layer 2) the Virtual Machine (where the LLG is running in containerized form) is connected to the SHLL’s physical network infrastructure through a Virtual Local Area Network (VLAN) following the IEEE 802.1Q specification.

At Network level (OSI layer 3) the LLG’s VM is configured with dual network; one subnet is used to access the livinglab devices, each with its own IP address; another subnet makes the LLG present in a De-Militarized Zone (DMZ) network, which is populated with other protected servers. A Virtual Private Network (VPN) can be used for management access of the nodes in both networks, the subnetwork of the VPN is independent from the other two therefore it is routed for access.

At Transport Level (OSI layer 4) the VM that hosts the LLG’s software stack is protected with an firewall, only port 443 is opened for public HTTPS connection. At this point all LLG’s services are directly available only in the DMZ or VLAN locally in the data center, or the SHLL. The containerization platform offers additional protection by providing local area network, and the associated routing, for the LLG’s micro-service architecture. At Session Level (OSI layer 5) incoming and outgoing connections are based on the HTTPS protocol, i.e., HTTP over TLS which includes valid certificates provided by Let’s Encrypt service [39] through the ACME protocol. This ensures communication uses state-of-the-art confidential point to point encryption. The transmitted information is only readable between each public client and the LLC’s endpoint.

In the LLG’s software stack, The application level (OSI layer 7) the passwords are stored at the database as hashed strings encrypted with sha256 algorithm (i.e., 256 bits Secure Hash Algorithm) following Linux system’s password shadow approach [40]. Furthermore, password fields are escaped and validated, avoiding unwanted string injection in MongoDB queries. Additionally. Moreover, authentication and authorization mechanisms implemented are based on JSON Web Token (JWT) standard. A client, before starting any interaction with the devices, must be authenticated and at each service call, needs to provide the access token in the message header which is approved for authorization, being token maximum lifetime 24 h.

**Figure 5:**
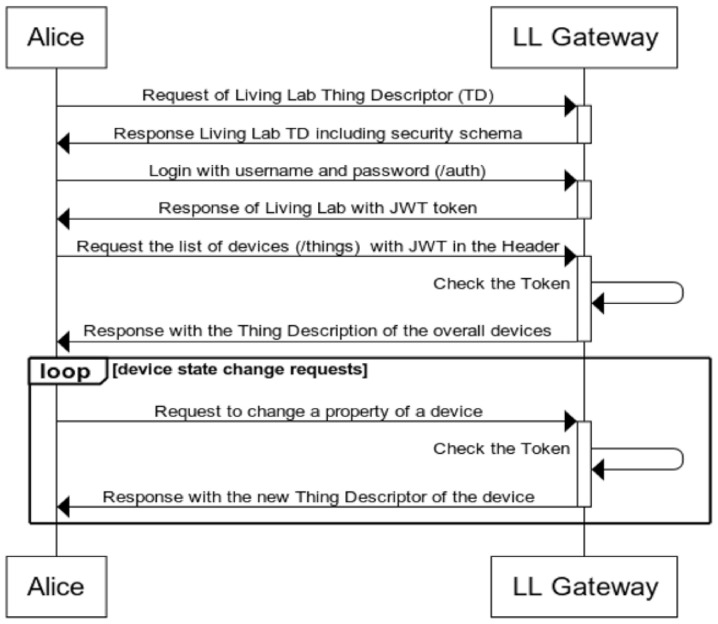
Authorization and Authentication with LLG.

Figure 5 shows the sequence diagram on how to interact with the smart gateway in order to be able to access the devices of the SHLL. The LLG provides a Thing Descriptor (TD) to the clients (e.g., Alice), the TD describes 2 endpoints: the /auth endpoint that a client needs to use in order to get credentials in JWT format [34] (the OAuth 2.0 authorization server role), and the /things endpoint that lists the accessible devices (the resource server role in OAuth 2.0 specification [41]) to the client, for which the JWT bearer header is required.

**Figure 6:**
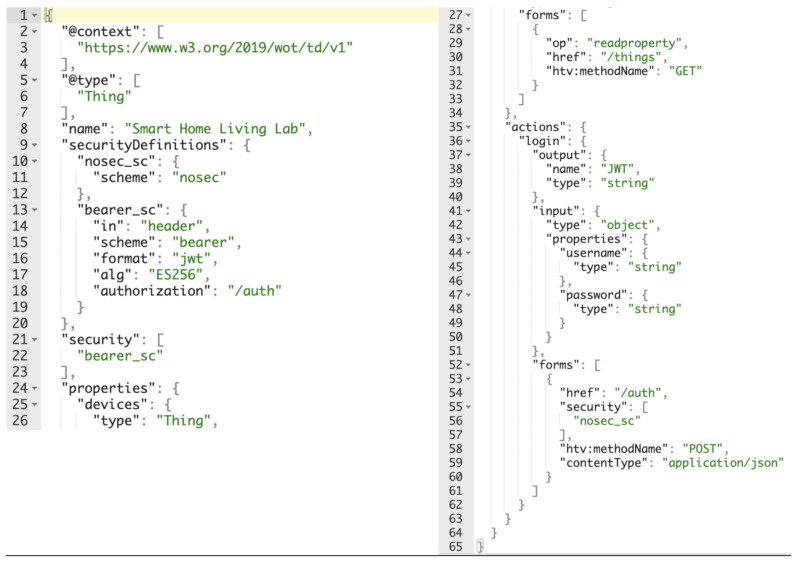
Living Lab Gateway Thing Descriptor.

Figure 6 shows the code snippet where the TD of the Lab is presented. As it can be appreciated, human readability is one of WoT Description’s key features, and several aspects can be inferred by looking at the snippet:

- A Thing that is the “Smart Home Living Lab” that supports 2 security access definitions. The first one, is an open access (nosec_sc), the second one (bearer_sc) is a bearer authentication based on JWT. The default access way is the bearer authentication.
- This “Smart Home Living Lab” has a property “devices” that is a Thing and an action that is “login”.
- The device property is read only, protected behind bearer authentication based on JWT and only accessible with an HTTP GET request to the relative path /things.
- The login action is an open access functionality that includes an input and an output object. The input is a JSON object formed by two string properties: a username and a password. The output is a JSON object formed by one string property, JWT. This functionality is accessible with an HTTP POST request to the relative path /auth where the body of the request is the input object while the expected result is the output object.

The TD describes the SHLL’s devices discovery process, specifying a communication with the LLG requiring the use of a token to access the JSON-LD device descriptions. These JSON-LD also speciy the WoT context vocabulary definition [42] and the security schema required to access and manage each device, i.e., the same JWT needs to be presented and validated before every request is processed.

### 3.3. From Datapoints to Things to Ontologies

The LLG provides semantic interoperability with standard vocabularies and ontologies formatted as JSON-LD contexts. Using Calimero Library enables users to change the value associated to a KNX^®^ datapoint address on the KNX^®^ infrastructure. Calimero library already provides a certain level of abstraction, yet it lacks the association between the KNX^®^ group address and the device’s definition. In previous work [17] it was already provided a mapping of KNX^®^ datapoints on URI following a RESTful Richardson Maturity Model level 1 [43] so, in our approach, we aim at providing a compliance with a RESTful Richardson Maturity Model level 3 HATEOAS (Hypermedia as Engine of Application State).

**Figure 7:**
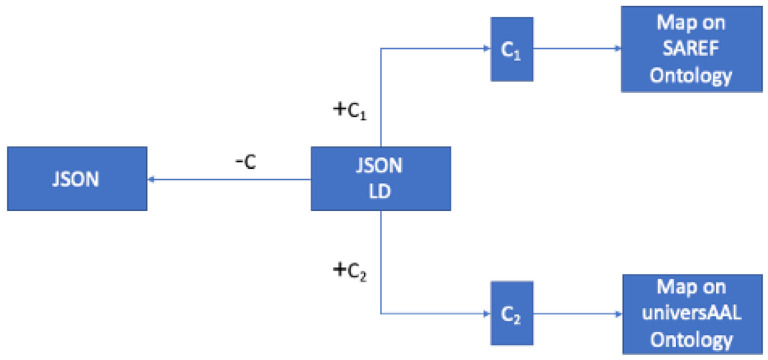
JSON-LD contexts mapping into context C1 and context C2.

As described in Section 2.1.7, in a Level 3 API (three-tier pattern) a resource not only describes the available actions to execute, but also describes the data itself. Certainly, property names such as “title” or “author” have no meaning to a computer, however, by using a shared vocabulary such as the one defined by Schema.org, machine-understandable meaning can be associated to data. Thus, when using self-descriptive data, it is possible to automate the interactions between systems with no human assistance. The process description to define context sensitive data mappings to bridge between two contexts with JSON-LD is shown in Figure 7, representing the JSON-LD that includes two contexts (+C1, +C2). If we remove the contexts (−C) we have just a JSON, but when we add different contexts (e.g., +C1 for SAREF, +C2 for universAAL) we can map data on different ontologies.

**Figure 8:**
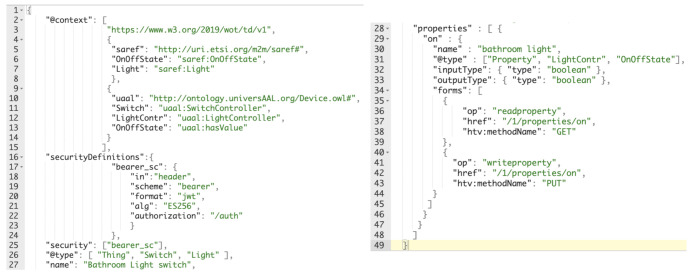
Thing description including SAREF and uAAL ontologies.

Taking an example from from the Smart House, Figure 8 describes the TD displayed by LLG of a *on/off* device (bathroom light) linked to SAREF and universAAL device ontology, as implemented:

This TD is describing the device named “Bathroom Light switch” as a Thing but also as a SAREF light and universAAL switch. It also has a property named “on” that is accessible through a bearer security schema and that is readable with an HTTP GET to the path /1/properties/on and writable with a HTTP PUT to the path /1/properties/on, sending the following data content: {“on”:true} or {“on”:false}.

### 3.4. Infrastructure and Deployment

The SHLL infrastructure is a premises components (KNX^®^-BUS and IP-KNX^®^) and cloud components hybrid deployment. This section describes how the LLG has been deployed in the cloud by using the Kubernetes engine, ensuring a reliable management of external requests of interaction with the SHLL.

The LLG is formed by a java application and a database in MongoDB, packaged in Docker images, which run in containers. The containers are then grouped in the same logical pod using kubernetes engine.

The MongoDB database container, deployed in the Kubernetes pod, uses persistent volumes and persistent volume claims to store data, being those independent of pod life-cycles and able to retain data by restarting, reprogramming, and even deleting pods. Additionally, the database requires a Persistent Volume to store data, while the Persistent Volume Claims make requests for storage, allowing a user to consume abstract storage resources with varying performance properties, delegating the management reliability, availability, bandwidth and other variables to the cloud provider.

**Figure 9:**
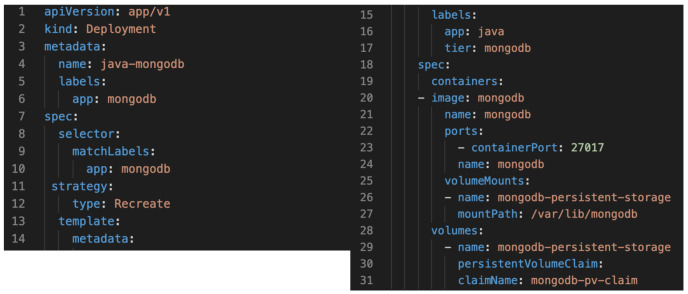
Kubernetes Manifest.

The following Kubernetes manifest shown in Figure 9 (used to create, modify and delete Kubernetes resources) describes single-instance MongoDB Deployment. The MongoDB container mounts the Persistent Volume at /var/lib/mongodb and then claims the provider for it.

**Figure 10:**
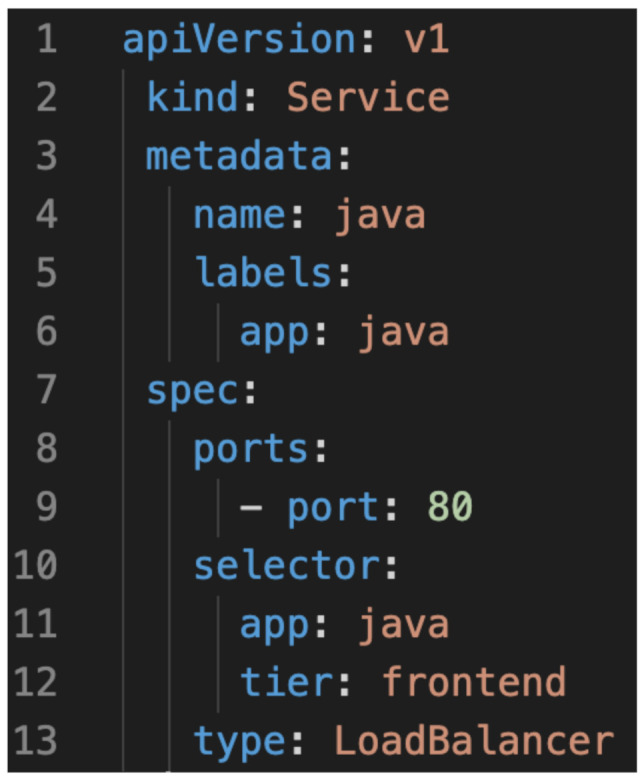
Single-instance JAVA deployment.

Figure 10 shows a manifest which describes the single-instance java Deployment. The java app will access the database deployment within the same pod and it is defined as a Service. The important point in the deployment of the java application is that the service is associated with a Load Balancer that delegates to the cloud provider the management of the availability of the service across different instance replicas of the servers.

By using Persistent Volume, Persistent Volume Claim and Load Balancer, LLG’s scalability, fault tolerance and availability is granted by the cloud provider that manages the Kubernetes cluster where the pod (that is instantiating the LLG) is running.

**Figure 11:**
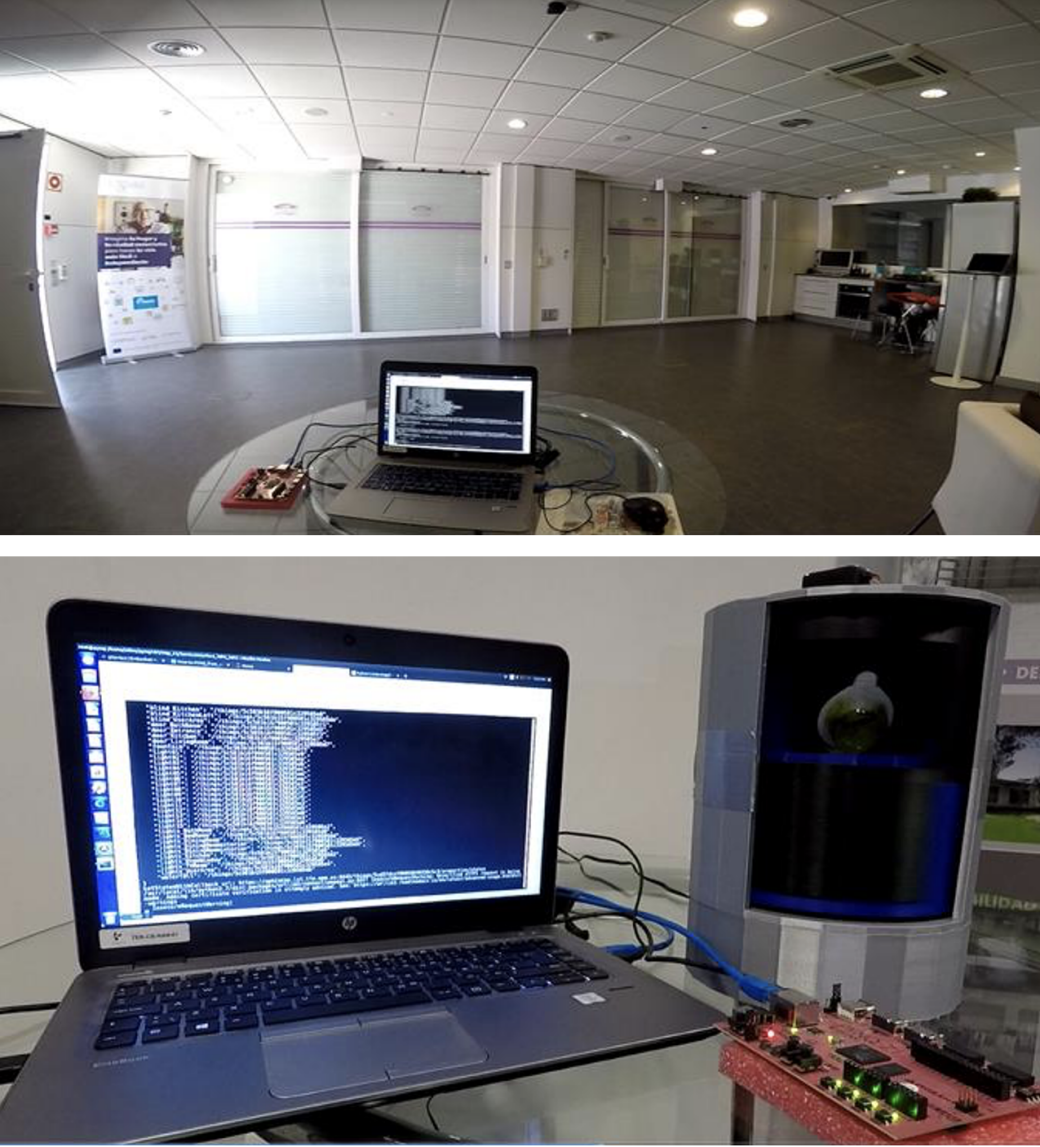
Field Programmable Gate Array controlling KNX^®^ devices (upper image) and an IoT device (lower image) via LLG.

Figure 11 illustrates the Scenario 1 tests performed at the Smart House Living Lab. In this case, an early version of the Field Programmable Gate Array controller is using the LLG to control different KNX^®^ devices (main door, lights and windows), executing activation sequences (as required by the Plan4Act project experiment). It also shows the interaction with an IoT device (the Smart Cabinet) to also perform movement sequences, such as switching compartments with different to show cooking ingredients.

Table 3 shows all cases tested with LLG and the results reported to the European Commission.

### 3.5. Response Time Comparison Test Results

Aiming to find out whether the LLG performance in response latency was comparable to direct HTTP loop, a paired test comparison was executed, creating *Direct_connection* and *LLG_connection* variables. The reason behind this choice was the local network’s bandwidth variability (induced by traffic). For this experiment, 500 response time samples were drawn in pairs (one of each method) and a paired *t*-test was performed, considering the null hypothesis as $H_{0}$: $\mu_{LLG}-\mu_{D}=0$ (as testing the difference between paired variables is equivalent to testing $\mu_{LLG}=\mu_{D}$) and alternate $H_{1}$: $\mu_{LLG}-\mu_{D}\neq 0$.

**Figure 12:**
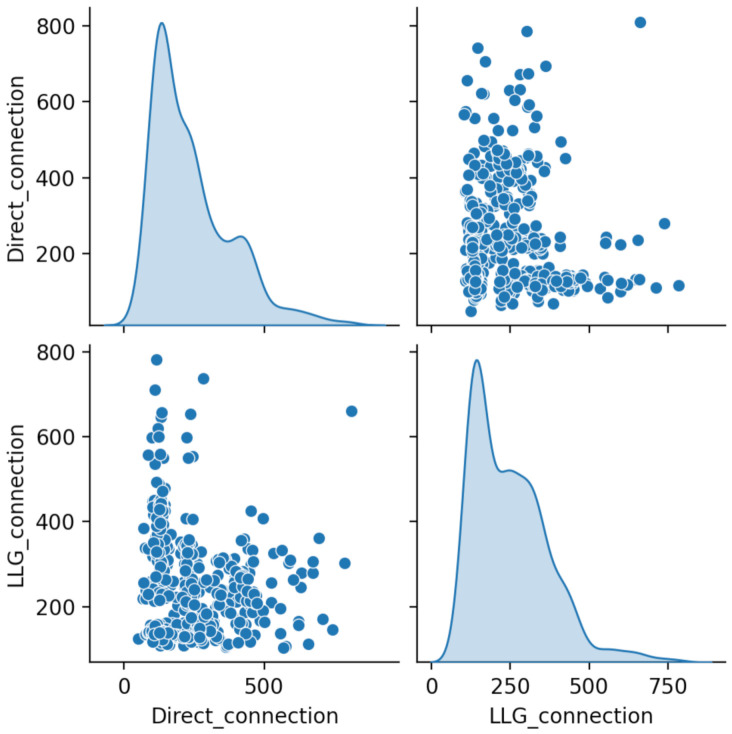
Direct_connection and LLG_connection scatter plots comparison.

A priori, a resemblance between distribution plots can be observed in Figure 12 but scatter plots present a vast amount of values laying far away from the trending region.

**Figure 13:**
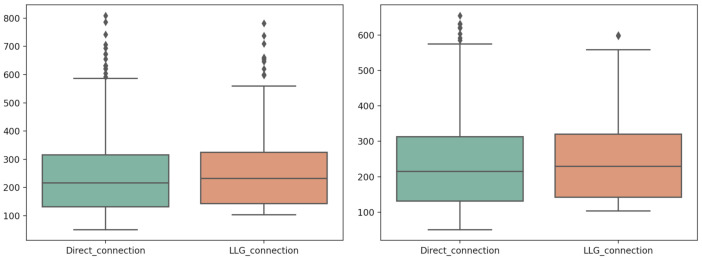
Dataset comparison (N = 500). Response time including outliers (**left**) and statistically removed outliers (**right**).

High variability induced by variable traffic load in our network can be appreciated in the boxplot diagrams shown in Figure 13, but this variability was mitigated by statistically removing outliers (z−score>3).

**Figure 14:**
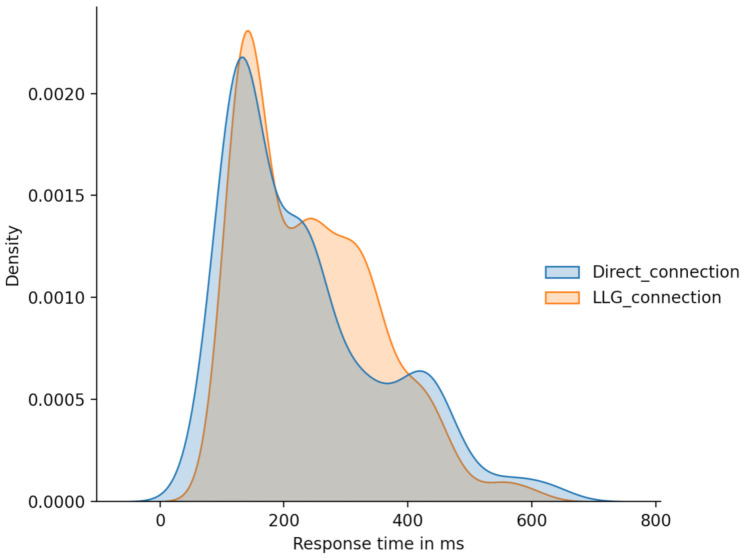
Direct_connection and LLG_connection distribution comparison.

Moreover, the apparent resemblance between distribution of both variables can be appreciated in Figure 14.

Finally, to find out whether similarity between both variable’s means, the *t*-test value were calculated for α=0.05. Particularly, $H_{0}$ can be rejected if $|t_{0}>|t_{\alpha /2,n-1}|$ (two-tailed test). For this test, the computed value for *t*-test statistic is $t_{0}=1.09638$, being $|t_{\alpha /2,n-1}|=1.960$, thus the null hypothesis cannot be rejected. Additionally, *p*-value computation resulted *p*=0.2734, confirming that we cannot reject the null hypothesis at the chosen level of significance. Other metrics worth reporting are the mean response time for each group: $\mu_{LLG}$ = 243.45 ms; $\mu_{D}$ = 234.35 ms. Also, to include a reference of the time consumed specifically by the communication protocol, the internal Smart Cabinet processing mean time was measured, resulting the mean time elapsed to process a request’s incoming characters and emmiting a response, $\mu_{SC}=37$ ms.

Finally, we can conclude that the mean response time is the same, either by using direct requests or passing through the LLG, confirming that our solution can be competitive as it does not add significant delay to the operation, yet including all above-mentioned benefits.

## 4. Discussion

The presented solution proposes versatility and flexibility for devices management in the home automation domain, as new devices can be injected and consequently operated through the LLG, as long as they have a thing description specifying the binding protocol over HTTP. An interesting feature to consider adding for a more automated device management (especially to populate the IoT devices list) could be the use of solutions for automated IoT onboarding, such as AIDE mechanism [44], which could help recognizing and auto-configuring surrounding devices avoiding manual mapping between devices and their digital twins.

A key LLG feature is the high level of abstraction provided attributed to the ontological data model which allows new device types to be easily adapted to the system, using the specification of JSON-LD Context. This is an example of semantic interoperability, where entities using difference reference models can interact due to semantic mapping and inference.

The proposed solution is extensible and scalable by design, as it can be extrapolated to other environments with the help of WoT standard, supposing no limitation in the amount or complexity of devices and services contained in the Things Descriptor. The deployment through Kubernetes system provides the LLG with capabilities such as scalability and traffic management. Although these capabilities are not required for our Smart House Living Lab, they may be fundamental for high performance demanding implementation such as smart buildings or campuses. As described above, interaction test were performed to measure the LLG’s incidence in response time against direct HTTP commands, and it was proven that the measured difference is actually not significant, even more considering the features offered by it.

Interestingly, the properties conferred by WOT standard allow the composition of more complex and powerful services by combining each device’s properties, data and available interactions, thus re-orienting and adding value to those elements and resources, revamping their original purpose with no extra costs.

Another interesting feature successfully implemented is the inclusion of universAAL devices ontology. It might not be as popular as other ontologies on the domain, for this reason we have mapped also the SAREF ontology, which has more support from the community and the European commission. However, both ontologies complement each other, where universAAL has deeper taxonomy of home automation sensors and actuators, SAREF focuses more on the process, adding extra versatility to the solution in terms of device description and usability.

The proposed approach in this article differs from the presented examples in several aspects. Compared to eWot, our solution uses JSON-LD instead of RDF for a more human readable description to describe all resources, relying on ontologies to describe the context to translate each one into a machine-understandable format.

Also our proposal does not use Software web enablement, as IoT-SIM, but rather implements a Secure, trustable and scalable API to deal with devices description, data and interoperability.

Regarding the model presented by the authors in [20], we consider that our solution provides a substantial simplification to resolve what they call User request description and User request resolutions (aimed at retrieving data or information of an entity or interacting with it), as our solution is based on the standard and extensively used HTTP protocol.

A key point in which the presented solution may be improved is its security. Even though high security standards implementation was not Plan4Act’s scope, user authentication and authorization was anyway ensured in order to protect important assets such as the SHLL and the devices. In fact, much of the security, scalability and trust features depend on the local deployment infrastructure, i.e., Controlled Network Management. The authentication and authorization proposed is embedded in the solution, allowing for the portability as-is to other compatible deployment. However, in the current state of art, security typically relies on other micro-services, while our approach makes authentication and authorization extensible by default, allowing for single sign on system or a more granular resources’ authorization. The OAuth 2.0 model enables the distribution of these services through different providers, effectively allowing externalization of security to other micro-services. In this line, using existing security management systems such as Keycloak could be explored. Keycloack already has a plethora of interesting features such as federated authentication, easily integrating single sign on systems or using other identity providers such as social networks and even being able to combine them. Arbitrary, but compatible with the standard, authentication and cybersecurity schemes can be used through this externalization. It would also manage, with a great granularity degrees, the authorization levels for each of the resources, and even doing so in a dynamic way (automatically assigning privileges to injected devices or changing them on run-time).

The evolution of the system could be extended to other domains. For instance, the LLG is primed to generate resources which could be used in machine learning and analytics engines to generate interesting and innovative services. In this sense, other more complex scenarios such as industries (e.g., to improve the process efficiency and safety conditions), smart cities (e.g., to optimize public service management)and healthcare systems could be the next challenges to be addressed, being the last one of high impact due to the straightforward inclusion of FHIR HL-7 [45], an ontology dedicated to healthcare records, images, wearable technology data and healthcare professional’s use. It could be possible to integrate sensible patient data with our solution and provide the means to foster better and faster diagnostics and healthier recommendations. As discussed above, for such implementations, especially those related to industry and healthcare given that they are a usual target for cyber attacks, more robust and sophisticated solutions such as those presented in [46,47] could be considered to be included in future upgrades. Moreover, by applying SRTIDE and DREAD methodology we found that our LLG has vulnerabilities (social engineering attacks), yet they constitute acceptable risks and can be mitigated by implementing modern authentication techniques such as the strong factor authentication mentioned. However, for the LLG it was decided not to use them, because in the WoT TD SecuritySchema object model, strong factor authentication is still not modelled. This implies that including strong factor authentication in our Thing Description would be at the expense of interoperability which is the main focus of our work.

Among the challenges (and lessons learnt), it is worth mentioning that when this development started, it was based on early WoT versions, which were being updated almost on a monthly basis, forcing our team to constantly update the code to cope with it and delaying our progress at the initial phase. Another significant challenge was the Kubernetes’ deployment. In terms of performance, Kubernetes system is optimal but it is very complex to deploy and may result in one of the most burdening overhead efforts. This fact for sure left room for improvements and we are exploring other systems such as Open Shift to simplify LLG’s deployment, as the knowledge and experience demanded to achieve a Kubernetes resilient deployment is considerable. On the other hand, in terms of validation, it would have been useful to quantitatively and qualitatively measure user’s experience while interfacing with LLG. Even though the validation phase was not in Plan4Act project’s scope, these metrics could help us to support the LLG’s true impact and help us determine the next steps. Finally, the integration of the mentioned technologies was a straightforward process for the SHLL, but this may not be always the case. Within our team, experts in ontologies (particularly SAREF and uAAL) had made previous progress and the SHLL devices were already described and little effort had to be made to develop and populate the Thing Descriptor.

## 5. Conclusions

Our results show that it was possible to access, consume and even add new devices (IoT) to the existing ones listed on KNX^®^ by using the LLG. This means that, at gateway level, there is no difference among KNX^®^ based devices and, to take our example of the air pollution sensor, they are presented through a common interface that is transparent to any client that want to use them.

It was possible to successfully expose the interaction model to clients’ applications in terms of properties, actions, and events, along with the associated data models and metadata, such as units of measure. Semantic descriptions of things were also achieved within the Thing Description, as it describes each device and their context of operation by implementing the standard ontologies, making this content usable and machine-understandable.

Communications metadata was also included in the Thing Descriptor, which describes how the client platform can access things, supporting the implemented protocols and standards. Moreover, security metadata was added to the description, stating what is needed for secure access to a thing. In this case, the Security schema (JSON Web Token Bearer) to be used in order to access to specific content that exposes how to access and interact with the Smart House resources.

Given the different deployment settings at the SHLL and the results presented, it is possible to state that the LLG achieved the expected results for device management and is viable to be implemented as a Smart Home resources management system, to overcome technology fragmentation in a scalable, trustable, and secure.

## Figures and Tables

**Table 1:** STRIDE analysis on the assets to be protected, in order to find the potential threats.

Asset	S	T	R	I	D	E
SHLL	Unauthorized physical access, disaster, through the Physical Devices					
Physical Devices	Unauthorized physical access, disaster, through the KNX^®^ Gateway					
KNX^®^ Gateway	Through VLAN					
VLAN	Unauthorized physical access, disaster.					
Thing Description	through LLG	through server	through DB	N/A	N/A	Through LLG
DB	DB misconfiguration or DB vulnerability	program verification violation	DB vulnerability	N/A	Request flooding on VLAN	ssh vulnerability and through VLAN
Server	OS Misconfiguration, OS vulnerability	Non authorized physical access, OS vulnerability	Log removal, SO vulnerability	N/A	OS vulnerability	SO or ssh vulnerability and through VLAN
Public access	TLS vulnerability	Firewall misconfiguration	TLS vulnerability	TLS vulnerability	Network flooding	TLS vulnerability
LLG	Social engineering	program verification violation	Limitted logs	through Public access	Resiliance violaiton	N/A (only one role)

**Table 2:** DREAD Classification for each Identified cybersecurity threat.

Attack Vector	D	R	E	A	D	Mitigation Action
Unauthorized physical access	H	L	L	H	L	Physical security (locked rooms, alarms, etc.)
Disaster	H	L	L	H	L	Off-site backups, Accepted risks
DB misconfiguration	H	M	L	M	M	Configuration review by Operation manager
DB vulnerability	H	H	L	H	L	Frequent software updates, Accepted risk
Program verification violation	H	L	L	H	L	Signed binaries
Request flooding on VLAN	L	L	L	H	L	Controlled Network management (Firewall, VPN, private VLAN), Accepted risk
ssh vulnerability and through VLAN	H	L	L	H	L	Frequent software updates, Controlled Network management (Firewall, VPN, private VLAN), Accepted risk
OS misconfiguration	L	L	L	H	M	Configuration review by Operation manager
OS vulnerability	H	L	L	H	L	Frequent software updates, Accepted risk
Log removal	L	H	L	L	L	Frequent backups, Accepted risk
TLS vulnerability	M	L	L	L	L	Frequent dependencies/software updates, Accepted risk
Firewall misconfiguration	M	L	L	H	M	Configuration review by Operation manager
Social engineering	H	L	M	L	L	Only experienced users, Accepted Risk
Limitted logs	L	M	L	H	L	Accepted Risk
Resiliance violaiton	M	M	L	H	L	Kubernetes with replication factor 3, Accepted risk

**Table 3:** List of test cases verifying interface between Plan4act’s FPGA controller and smart devices. KNX^®^ devices involved are: A-Main door; B-main lights; C-Bathroom door; D-Sliding window. IoT device: Smart Cabinet, being B1 or B2 commands for choosing upper or lower compartment, and C1 or C2 for choosing left one or right one.

Description	Target	Status
Proactive control for sequence A-B-C	Smart House	Pass
Proactive control for sequence A-B-D	Smart House	Pass
Proactive control for sequence B1-C1	Smart Cabinet	Pass
Proactive control for sequence B1-C2	Smart Cabinet	Pass
Proactive control for sequence B2-C1	Smart Cabinet	Pass
Proactive control for sequence B2-C2	Smart Cabinet	Pass
Sequential control for sequence A-B-C	Smart House	Pass
Sequential control for sequence A-B-D	Smart House	Pass
Sequential control for sequence B1-C1	Smart Cabinet	Pass
Sequential control for sequence B1-C2	Smart Cabinet	Pass
Sequential control for sequence B2-C1	Smart Cabinet	Pass
Sequential control for sequence B2-C2	Smart Cabinet	Pass
